# Developing a Naïve Bayes risk classification machine learning algorithm to predict high viral load in a low-resource setting

**DOI:** 10.1371/journal.pgph.0006373

**Published:** 2026-05-22

**Authors:** Laston Gonah, Trymore Murakwani

**Affiliations:** 1 School of Public Health, Faculty of Medicine and Health Sciences, Walter Sisulu University, Mthatha, South Africa; 2 Independent Researcher, Zimbabwe; PLOS: Public Library of Science, UNITED STATES OF AMERICA

## Abstract

Access to routine viral load (VL) testing for people living with HIV (PLHIV) remains limited in many low-resource settings. There is a need for pragmatic, data-driven tools that can proactively identify individuals at increased risk of virological failure. This study aimed to identify predictors of high viral load among PLHIV on antiretroviral therapy (ART) and to evaluate the performance of a Naïve Bayes classification algorithm using routinely collected clinical data. We conducted a retrospective case-control study using secondary clinical data from two public ART facilities in Makonde District, Zimbabwe. A total of 530 participants (156 cases with VL ≥ 1000 copies/mL and 374 controls) were included. Logistic regression was used to identify independent predictors of high VL. A Naïve Bayes classification model was developed using significant and clinically relevant predictors. Model performance was evaluated on the development dataset, and sensitivity, specificity, predictive values and overall accuracy were calculated. Independent predictors of high VL included being a child (adjusted OR 6.11, 95% CI: 2.12-9.31), adolescent/young adult (AOR 4.69, 95% CI: 1.83-7.54), single/non-partnered marital status (OR 2.01, 95% CI: 1.02-3.04), nondisclosure of HIV status (OR 2.56, 95% CI: 1.48-4.17), ambulatory functional status (OR 3.09, 95% CI 1.64-5.43), recent weight loss on two consecutive visits (OR 4.48, 95% CI: 2.11-8.03, p < 0.001), and ART duration <5 years (OR 1.78, 95% CI 1.15-2.63). The Naïve Bayes model demonstrated a sensitivity of 78.2% and specificity of 96.5%, indicating that the model may miss approximately one in five individuals (22%) with true high viral load, misclassifying them as low risk. The developed Naïve Bayes algorithm shows potential as a risk classification tool to support prioritization of VL testing in resource-limited settings. However, performance estimates represent apparent (development-only) performance and require validation in independent datasets before implementation.

## Introduction

By the end of 2024, an estimated 40.8 million people were living with HIV globally, of whom 31.6 million were receiving antiretroviral therapy (ART) [1]. As demonstrated in trials such as HPTN 052 and PARTNER study [2], effective ART enables people living with HIV (PLHIV) to achieve viral suppression, substantially reducing morbidity, mortality and risk of sexual transmission.

Viral load (VL) monitoring is the most accurate indicator of treatment success because it directly measures the degree of viral suppression achieved with ART [1,3]. The World Health Organization defines VL ≥ 1000 copies/mL as virological failure requiring clinical intervention [4]. In high-income settings, VL monitoring is typically performed more frequently during ART initiation and stabilization phases, with longer intervals for stable patients, as reflected in guidelines such as those from the U.S. Department of Health and Human Services [5].

In many sub-Saharan African settings, including Zimbabwe, universal and timely VL testing is challenged by laboratory constraints, reagent stock-outs, transport delays, and infrastructure limitations [6–8]. Although national viral suppression rates are high, maintaining comprehensive VL coverage requires substantial laboratory capacity [9]. As a result, many health systems have adopted targeted VL testing, where testing is limited to clients showing clinical or immunological signs of treatment failure, which risks missing asymptomatic patients with rising VLs [10,11]. Developing evidence-based risk classification models for identifying individuals at highest risk of virological failure could support more efficient allocation of testing resources and improve clinical outcomes.

Machine learning approaches offer opportunities to identify complex patterns within routine clinical data. The Naïve Bayes classifier is particularly suitable in low-resource settings because it is computationally efficient, interpretable and performs well with categorical predictors [12,13]. This study aimed to identify predictors of high VL and evaluate the performance of a Naïve Bayes model using routinely collected ART program data from two ART sites in Zimbabwe.

## Methods

### Ethics statement

This study utilised retrospectively collected, routinely recorded clinical data. Since the research involved secondary analysis of de-identified programmatic records, individual informed consent was waived by the approving ethics committees. All data were anonymised prior to analysis to ensure confidentiality and privacy. The study was conducted in accordance with the Declaration of Helsinki and received ethical approval from the Medical Research Council of Zimbabwe (MRCZ) and the University of KwaZulu-Natal Biomedical Research Ethics Committee (UKZN BREC; Ref No: BE086/23) prior to data extraction.

### Study design and setting

This retrospective case-control study used routinely collected data from two public ART facilities in Makonde District, Mashonaland West Province of Zimbabwe, namely, Chinhoyi Provincial Hospital and Chikonohono Clinic. The study sites are the two highest-volume public ART facilities in the district, collectively serving approximately 40% of all ART clients in the province [14]. Data were extracted for clients with VL results collected within 12 months prior to 31 December 2024.

### Study population, data sources and sample size

Eligible participants were PLHIV of any age with a documented VL result within the defined timeframe. Cases were defined as VL ≥ 1000 copies/mL; controls were defined as VL < 1000 copies/mL [1,4]. Unmatched controls were systematically sampled at a ratio of 2:1 (controls: cases) from the same facilities and time period, to achieve proportional representation.

Data were extracted from three primary sources: (i) the OI/ART Care Booklet which is the main patient record documenting demographic, clinical, and treatment data; (ii) the Viral Load Register, which is used to identify clients with recent VL results; and (iii) the Electronic Patient Monitoring System (ePMS), which compiles historical data from the OI/ART Booklet. Only routinely recorded, objectively verifiable data were used to minimize recall and reporting bias.

Data for clients: (a) whose VL samples were older than 12 months; (b) who had undergone enhanced adherence counselling (EAC) before a repeat VL test; (c) who had switched to a dolutegravir-based first-line regimen before data collection; and (d) who had defaulted (patients missing ART visits beyond the national default definition of >90 days without documented return), were excluded to address confounding and reduce biased model performance.

Sample size was estimated using the Fleiss formula [15] with continuity correction, assuming 80% power, 95% confidence level, a 1:2 case-control ratio, and an expected odds ratio of 2 for key exposures. A minimum of 335 participants (112 cases and 223 controls) was required. The final dataset included 530 participants (156 cases and 374 controls).

### Study variables and statistical analysis

Extracted variables included age, sex, marital status, educational level, HIV disclosure status, employment status, functional status, pregnancy/breastfeeding status, duration on ART, weight change over two consecutive visits and prophylactic therapy use (cotrimoxazole, isoniazid).

Descriptive statistics and logistic regression analyses were performed using STATA/SE 12.0. Variables with clinical relevance and statistical significance (p < 0.05) were included in the predictive model. Data cleaning and descriptive analyses were performed using STATA/SE 12.0. Univariate and bivariate analyses summarized proportions and assessed associations (χ² tests for categorical variables). Logistic regression produced crude and adjusted odds ratios (ORs) with 95% confidence intervals (CIs).

### Predictive model

The final modelling was conducted in Python software (Scikit-learn library), and Naïve Bayes classifier was developed using selected predictors. The algorithm applies Bayes’ theorem under an assumption of conditional independence among predictors, simplifying computation while retaining strong predictive performance [13,16]:


$$\begin{array}{c} p\left(High|Class\right)=\;\frac{p\left(Class|High\right)*p(High)}{p(Class)} \end{array}$$

Where, p(High)is the overall probability of high viral load, p(Class)is the probability of a specific combination of characteristics (normalizing constant), p(High∣Class)is the posterior probability, and p(Class∣High)is the likelihood. Predictors were selected based on logistic regression significance (p < 0.05) and clinical relevance [16]. Correlations between candidate predictors were examined prior to model development to minimize violations of the conditional independence assumption inherent to the Naïve Bayes framework.

Model performance was evaluated on the same dataset used for model development. For model evaluation, we calculated: (i) sensitivity - proportion of true high VLs correctly identified; (ii) specificity - proportion of true suppressed VLs correctly classified; and (iii) positive predictive value (PPV) and negative predictive value (NPV). Reported sensitivity, specificity, PPV, NPV and overall accuracy therefore represent apparent (resubstitution) performance. Model accuracy was assessed using a confusion matrix comparing predicted versus actual outcomes. Because this was a case-control study with oversampling of high-VL cases, sensitivity and specificity are emphasized as primary performance measures. Predictive values should be interpreted cautiously as they depend on prevalence.

## Results

### Participant characteristics

A total of 530 participants were included in the analysis, comprising 156 (29.4%) cases with VL ≥ 1000 copies/mL and 374 (70.6%) controls with VL < 1000 copies/mL. The mean age was 39.8 years (SD = 12.7), and 62% were female. As presented in **Table 1**, children and adolescents/young adults (0–24 years) accounted for 24% of cases versus 7% of controls (p < 0.001). Viral load did not differ by sex (p = 0.85), but participants who had not disclosed their HIV status were twice as likely to have a high VL (p < 0.001). Being single/widowed/divorced was associated with higher VL compared to married participants (p < 0.001), and ambulatory clients had greater prevalence of high VL than those classified as “working” (16% vs 6%, p = 0.02). Recent weight loss was strongly linked to high VL, observed in 49% of affected participants versus 22% among those without weight loss (p < 0.001).

**Table 1:** Participant Characteristics (Cases vs Controls).

Variable	Cases (n = 156)	Controls (n = 374)	Total (N = 530)	p-value
**Age group (years)** 0-12 13-24 25-49 50+	12 (7.7%) 25 (16.0%) 96 (61.5%) 23 (14.7%)	7 (1.9%) 19 (5.1%) 266 (71.1%) 82 (21.9%)	19 (3.6%) 44 (8.3%) 362 (68.3%) 105 (19.8%)	< 0.001
**Sex** Female Male	95 (60.9%) 61 (39.1%)	231 (61.8%) 143 (38.2%)	326 (61.5%) 204 (38.5%)	0.93
**HIV status disclosure** Yes No Undocumented	63 (40.4%) 39 (25.0%) 54 (34.6%)	286 (76.5%) 69 (18.4%) 19 (5.1%)	349 (65.9%) 108 (20.4%) 73 (13.7%)	< 0.001
**Marital status** Single/ Widowed/Divorced Married	85 (16.5%) 71 (58.7%)	141 (9.1%) 233 (62.3%)	221 (11.5%) 304 (66.5%)	<0.001
**Functional status** Working Ambulatory Treatment supporter	82 (81.2%) 16 (15.8%) 3 (3.0%)	111 (88.1%) 7 (5.6%) 8 (6.3%)	193 (85.0%) 23 (10.1%) 11 (4.8%)	0.02
**Change in weight (recent 2 visits)** Gained weight Lost weight No change	31 (27.7%) 55 (49.1%) 26 (23.2%)	101 (54.6%) 40 (21.6%) 44 (23.8%)	132 (44.4%) 95 (31.7%) 70 (23.9%)	< 0.001

**Note:** Percentages rounded to one decimal place. Totals vary slightly due to missing data (< 3%).

### Predictors of high viral load

Multivariable logistic regression identified several significant predictors of high VL (**Table 2**). Children (0–12 years) and adolescents/young adults (13–24 years) had markedly higher odds of high viral load (AOR 6.11 and 4.69, respectively) compared to those ≥50 years. Single status (AOR 1.93), non-disclosure of HIV status (AOR 2.56), ambulatory functional status (AOR 3.09), recent weight loss (AOR 4.48) and ART duration <5 years (AOR 1.78) were also independently associated with high VL. Pregnancy or breastfeeding showed a non-significant trend toward higher VL (AOR 2.07).

**Table 2:** Multivariable Logistic Regression Analysis of Factors Associated with High Viral Load (VL ≥ 1000 copies/mL).

Variable	Adjusted Odds Ratio (OR)	95%CI	p-value
Age group (ref: ≥ 50 years) 0-12 years 13-24 years 25-49 years	6.11 4.69 1.29	2.12-9.31 1.83-7.54 0.87-1.90	<0.001 <0.001 0.18
Marital status (ref: Married) Single/Widowed/Divorced	2.01	1.02-3.04	0.01
HIV status disclosure (ref: Disclosed) Not disclosed	2.56	1.48-4.17	<0.001
Functional status (ref = Working) Ambulatory	3.09	1.64-5.43	<0.001
Change in weight (ref = Weight gain) Weight loss	4.48	2.11-8.03	<0.001
Pregnant/Breastfeeding (ref = No)	2.07	0.92-3.13	0.09
Duration on ART (ref: ≥ 5 years) <5 years	1.78	1.15-2.63	0.02

### Model performance

The Naïve Bayes classifier demonstrated robust discriminatory performance in identifying individuals with high viral load (VL ≥ 1000 copies/mL). The model achieved a sensitivity of 78.2% and a specificity of 96.5%, indicating good overall capacity to distinguish between high VL and virologically suppressed individuals (**Table 3**). The positive predictive value (PPV) was 90.4%, while the negative predictive value (NPV) was 91.4%, reflecting strong predictive performance within the observed sample. Overall classification accuracy was 91.1%.

**Table 3:** Apparent Performance of the Naïve Bayes Model for Predicting High Viral Load (VL ≥ 1000 copies/mL).

Performance Metric	Estimate (%)
Sensitivity (True Positive Rate)	78.2
Specificity (True Negative Rate)	96.5
Positive Predictive Value (PPV)	90.4
Negative Predictive Value (NPV)	91.4
Overall Accuracy	91.1

A sensitivity of 78.2% implies that approximately 22% of individuals with true high VL were misclassified as low risk, whereas the high specificity (96.5%) indicates strong performance in correctly identifying individuals who were virologically suppressed. These performance estimates represent apparent performance within the analysed dataset. No projections regarding reductions in viral load testing are made, as such implications would depend on underlying population prevalence, implementation thresholds, and prospective external validation.

## Discussion

This study developed and evaluated a Naïve Bayes risk classification machine learning algorithm to predict high viral load (VL ≥ 1000 copies/mL) among PLHIV receiving ART in a low-resource Zimbabwean setting. Several independent predictors of high VL were identified, including younger age (0–24 years), nondisclosure of HIV status, single marital status, ambulatory functional status, recent weight loss and shorter ART duration (<5 years). The resulting classification model demonstrated good apparent discriminatory performance, with sensitivity of 78.2% and specificity of 96.5%.

The strong association between younger age and virological failure is consistent with existing evidence that children, adolescents and young adults often experience greater adherence challenges due to developmental, psychosocial and structural factors. Inadequate social support, transition gaps from paediatric to adult care, treatment resistance due to denial and identity incorporation struggles, stigma-related avoidance, low health literacy or treatment insight and treatment fatigue may contribute to suboptimal viral suppression in these groups [6,7,17–21]. Similarly, nondisclosure of HIV status emerged as an independent predictor of high VL, further reinforcing the role of social support and enhanced social acceptance in sustaining long-term adherence [22,23]. Association of single or non-partnered marital status with high viral load likely reflects the role of intimate social support in ART adherence and viral load outcomes, with married individuals potentially benefiting from stronger or sustained social, emotional, and resource support from their partners [24]. Further studies employing granular analyses are required to reveal the mechanisms through which socio-demographic factors influence ART outcomes and broader quality-of-life domains.

Clinical markers such as recent weight loss and ambulatory functional status were also strongly associated with high VL and possible treatment failure. These findings highlight the continued significance of simple routine anthropometric and functional measures in monitoring ART outcomes and identifying individuals at risk of treatment failure in resource-limited settings [25,26]. In underserved settings where access to laboratory services is constrained, anthropometric and functional assessments remain important components of patient monitoring [25,26]. The association between shorter ART duration and high VL may reflect early-phase adherence instability, incomplete immune recovery, or challenges in treatment optimization during the first years of therapy [27].

In this study, the Naïve Bayes model demonstrated high specificity, indicating strong ability to correctly identify individuals who were virologically suppressed. Sensitivity analysis found that approximately one in five individuals with true virological failure would be misclassified by the model as low risk. However, these performance estimates require validation, as they represent apparent (development-only) performance and must be interpreted cautiously. It is important to note that no projections regarding reductions in VL testing can be made from this study, as such implementation outcomes would depend on underlying prevalence, chosen risk thresholds and prospective validation. Nonetheless, the findings demonstrate that routinely collected programmatic data can be leveraged to support risk classification in low-resource ART programs. Distinct from most prior predictive studies conducted in highly resourced settings or using complex modelling frameworks, this study deliberately applied a computationally simple and interpretable algorithm. The Naïve Bayes approach is well suited to routine health systems due to its efficiency and transparency, making it potentially feasible for integration into electronic patient monitoring systems in similar contexts [12,13,16].

### Study limitations

There are important limitations that should be considered when interpreting the findings of this study. Model performance was evaluated on the same dataset that was used for development and therefore reflects apparent (resubstitution) performance. This approach likely overestimates true predictive accuracy, therefore, external validation in independent or temporally distinct datasets is essential before clinical or programmatic implementation.

Again, the retrospective case-control design, while appropriate for identifying predictors, does not reflect true population prevalence of high VL. Consequently, predictive values should be interpreted cautiously, and sensitivity and specificity are emphasized as primary performance metrics.

Also, reliance on routinely collected secondary data introduces the possibility of incomplete or inconsistently recorded variables. Behavioural and psychosocial factors such as adherence experiences, stigma intensity and social support could not be comprehensively captured in this study and must be considered in efforts to improve predictive performance in future models.

Notably, the study was conducted in two high-volume ART facilities within a single province, which may limit generalizability to other geographic, rural, or lower-volume settings. Additionally, exclusion of defaulters and individuals who had undergone enhanced adherence counselling may restrict applicability to the broader population of PLHIV.

Despite these limitations, the study represents a preliminary attempt to demonstrate how routinely available data can inform risk classification in resource-constrained health systems such as Zimbabwe.

## Conclusion

This study provides preliminary evidence within the Zimbabwean context that a Naïve Bayes risk classification machine learning algorithm can identify individuals at increased risk of high viral load using routinely collected clinical data. The model demonstrated high specificity and moderate sensitivity, suggesting potential utility as a prioritization tool to support targeted viral load testing rather than as a replacement for universal monitoring. Since performance estimates from this study represent apparent development-phase accuracy, external validation is required before implementation. Similar future research efforts should incorporate behavioural and psychosocial factors, evaluate model performance in independent cohorts, assess calibration and decision thresholds, and explore integration within electronic health systems. If validated prospectively, simple and interpretable predictive algorithms such as this may support more efficient allocation of limited clinical and laboratory resources, earlier identification of treatment failure and strengthened programmatic monitoring in HIV care settings.
